# Observation of the validity of the upper lip bite test in predicting difficult intubation

**DOI:** 10.1038/s41598-023-49642-9

**Published:** 2023-12-13

**Authors:** Xinyuan Tang, Zhiyuan Dong, Jianling Xu, Pingping Cheng, Mingfang Wang, Bin Wang, Xiaogan Jiang, Weidong Yao

**Affiliations:** 1 Department of Anesthesiology, The First Affiliated Hospital of Wannan Medical College, Wuhu, Anhui China; 2 Anhui Province Clinical Research Center for Critical Care Medicine (Respiratory Disease), The First Affiliated Hospital of Wannan Medical College, Wuhu, Anhui China

**Keywords:** Medical research, Risk factors

## Abstract

The upper lip bite test (ULBT) is considered an effective method for predicting difficult airways, but data on the ULBT for predicting difficult tracheal intubation are lacking. This study aimed to examine the clinical utility of the ULBT in predicting difficult endotracheal intubation. We conducted an observational case-cohort study of adult patients undergoing elective surgery and requiring endotracheal intubation for general anesthesia. Difficult airway assessment was performed on the recruited patients before the operation, including the ULBT, mouth opening, thyromental distance, modified Mallampati test, and body mass index. The primary outcome was the incidence of difficult tracheal intubation. The receiver operating characteristic curve analysis was used to compare the performance of variables in predicting difficult tracheal intubation. We successfully recruited 2522 patients for analysis and observed 64 patients with difficult tracheal intubation. When predicting difficult tracheal intubation, grade 2 ULBT had a sensitivity of 0.75 and a specificity of 0.54, and grade 3 had a sensitivity of 0.28 and a specificity of 0.75. Compared with mouth opening, the area under the receiver operating characteristic curve of the ULBT was lower in predicting difficult tracheal intubation (0.69 [95% confidence interval: 0.67–0.71] vs. 0.84 [95% confidence interval: 0.82–0.87], *P* < 0.05).

*Clinical Trials Registry*: ChiCTR-ROC-16009050, principal investigator: Weidong Yao.

## Introduction

Difficult airways present a significant risk to patients and require proactive management by anesthesiologists, intensive care physicians, and emergency physicians^1–3^. Difficult tracheal intubation is rare but serious in airway management and requires more attention from clinicians. Early screening can effectively identify patients at risk for difficult intubation^4^, but unfortunately, current screening methods for difficult airways are not accurate enough^2,5^. Khan et al.^6^ reported the upper lip bite test (ULBT) method for difficult airway screening. Several studies showed that the predictive power of this method was clinically significant for difficult laryngoscopy^7–12^, and reported that the ULBT may outperform other commonly used methods in predicting difficult airways. Despite doubts^13–16^, in review articles of Detsky et al.^2^ and Heidegger et al.^5^, the authors suggested that the ability of the ULBT to predict difficult airways is worth emphasizing. However, it should be noted that there are insufficient clinical data on the efficacy of the ULBT for predicting difficult airways. First, the sample size of any single study is relatively small, mostly between 200–500 cases^6–13,17–21^. Considering the 2–3% incidence of difficult intubation, these studies lack the statistical power to predict difficult intubation. Furthermore, in those studies, the definition of a difficult airway was based on difficult laryngoscopy, lacking data and evidence to predict difficult intubation. Based on data predicting difficult laryngoscopy, generalizing to difficult tracheal intubation, or even all difficult airway types, would be inappropriate and may give a false clinical impression. The purpose of this study was to observe the clinical utility of the ULBT in predicting difficult intubation.

## Method

With the approval of the ethics committee of Yijishan Hospital of Wannan Medical College, we conducted a prospective case-cohort observational study (the clinical trial was registered prior to patient enrollment, Clinical Trials Registry: ChiCTR-ROC-16009050, principal investigator: W.Y., date of registration: August 19, 2016). We recruited patients who required general anesthesia and intubation at the hospital from September 2016 to August 2018. The inclusion criteria were as follows: (1) patients needing elective surgery, with an American Society of Anesthesiologists physical status grade 1–3, and aged 18–90 years old; (2) patients without an airway injury or infection; and (3) patients without head and face tumors, anatomical deformities, missing incisors, or subglottic airway stenosis. The exclusion criteria were as follows: (1) patients who did not need laryngoscopy; (2) patients who could not tolerate endotracheal intubation; (3) patients who needed to be awake for intubation; and (4) patients who refused to participate in the study. All patients signed written informed consent forms.

### Airway assessment

All included patients underwent preoperative difficult airway screening assessment. In the ULBT, the patient was asked to bite their upper lip with their lower incisors as much as possible. Grade 1: If the skin of the upper lip is bitten; grade 2: if the mucosa of the upper lip is bitten; grade 3: if the upper lip cannot be bitten. Grades 2 and 3 were deemed difficult airway predictors^6^. In the mouth opening measurement method, the patient was asked to open their mouth as far as possible and the distance between the upper and lower incisors was measured. A distance < 4 cm was deemed a difficult airway predictor^22^. In the modified Mallampati test method^23,24^, the patient was asked to sit upright with their head in the center, mouth opened as wide as possible, and their tongue stuck out as far as possible. The patient was not required to pronounce any words, and the structure of the pharynx was observed. Grade 3 or 4 was deemed a predictor for difficult airways. In the thyromental distance measurement method, the patient laid in the supine position, making a “sniffing” face, and the distance from the tip of the chin to the notch of the thyroid cartilage was measured. A thyromental distance < 7 cm was deemed a predictor for difficult airways^22^.

Five investigators were responsible for preoperative airway assessment. They all have more than 3 years of clinical anesthesia experience and have been trained for research projects. To reduce study bias, investigators who performed preoperative airway assessments withheld their assessments from the responsible anesthesiologists. The responsible anesthesiologists performed airway assessment and made independent decisions by themselves.

### Outcomes of observation

After the patient entered the operating room, the patient was monitored and receives inhaled oxygen. In this investigation, general anesthesia was induced following a consistent and standardized protocol, which entailed administering midazolam at 0.03 mg/kg, sufentanil at 0.5 µg/kg, propofol between 1–2 mg/kg, and rocuronium at a dose of 0.9 mg/kg. Endotracheal intubation was performed by resident physicians or attending physicians who had more than 3 years of experience in administering anesthesia. Approximately 3 min after injection of neuromuscular blocking agents, endotracheal intubation (The inner diameter of the endotracheal tube is 7.0–8.0 mm, TUOREN Group Ltd. China) under ordinary laryngoscopy (Macintosh blade, 3 or 4#) was performed. A stylet was used to help shape the tube. The results of laryngoscopy and tracheal intubation were recorded. In this study, we engaged 42 anesthesiologists to conduct tracheal intubations, encompassing both attending physicians and residents licensed in anesthesiology. Each anesthesiologist had completed a minimum of three years of standardized postgraduate residency training in anesthesiology.

The primary outcome was difficult intubation, defined as > 2 intubation attempts, an intubation time more than 10 min or the need for a different intubation tool^5,25^. The secondary outcome was difficult laryngoscopy, defined as a laryngoscopy Cormack-Lehane level^24^ of 3 or 4.

### Security

If the anesthetist encountered a difficult airway, the corresponding guidelines should be followed. To ensure the efficacy of mask ventilation, the mask ventilation time between different intubation attempts should be greater than 1 min, and the oxygen saturation should be above 95%. Different attempts may have required changes in equipment or endotracheal intubation providers. Adequate alternative intubation tools, such as video laryngoscope, intubation light stick, fiberoptic bronchoscope, laryngeal mask and preparation for awake endotracheal intubation, should be prepared for all patients.

### Statistical analysis

SPSS software version 16.0 (SPSS, Chicago, IL) and MedCalc version 12.7 (MedCalc Software, Mariakerke, Belgium) were used to assist in the statistical analysis. Shapiro Wilk test was used for distribution normality test. The measurement data are displayed as the means (standard deviations) or median (interquartile range, IQR) when appropriate and the categorical variable data are presented as numbers. Statistical values are presented with their 95% confidence intervals (95% CI). Each evaluation parameter in difficult airway and non-difficult airway was compared by t test, chi-square test, Fisher’s exact probability, or nonparametric test as needed. Univariate regression analysis and receiver operating characteristic curve analysis were used to calculate the performance of variables in predicting difficult airways. The area under the receiver operating characteristic curves (AUCs) of different indicators for predicting difficult airways were compared using a nonparametric test^26^. Variables with significant differences in univariate analysis were included in collinearity analysis and multivariate logistic regression analysis (stepwise method), and retained variables were considered independent risk factors. According to the previous data, with a two-tailed test, α < 0.05, the statistical power is 0.9. To observe a difference of 0.2 in the AUC between upper lip biting grading and other methods for predicting difficult intubation, the required sample size is approximately 1800 cases, and the goal of this study was to include more than 2000 cases.

### Ethical approval

This study was approved by the Ethics Committee of Yijishan Hospital of Wannan Medical College and follows the Declaration of Helsinki. Written informed consent was obtained from participating patients.

## Results

**Figure 1:**
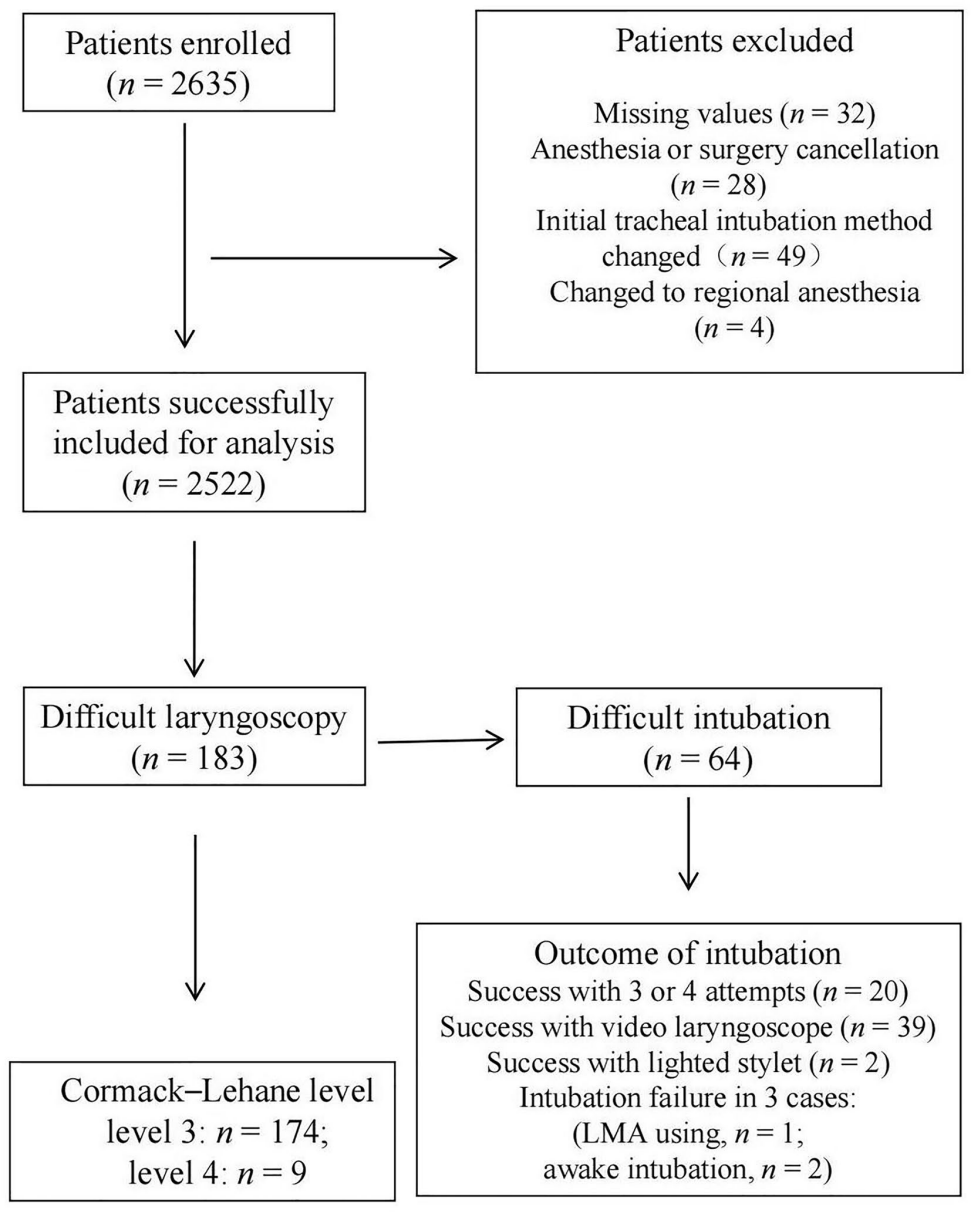
Study flow chart.

A total of 2635 patients were recruited during the study period; among them, 113 patients were excluded (Fig. 1). The general characteristics of the patients are listed in Table 1. In the comparison between patients with and without difficult intubation, the difference in ULBT results was significant (Table 1).

**Table 1:** Comparisons of patients with and without difficult intubation.

Variable	Difficult intubation (Yes) n = 64	Difficult intubation (No) n = 2458	*P* value*
Sex (Male/Female, n)	30/34	1212/1246	0.701
Age (y)^#^	55(49–67)	50(39–60)	< 0.001
Body mass index (kg/m^2^)^#^	24.0(21.5–25.7)	22.9(20.8–25.1)	0.152
ULBT > 1 grade (Yes/No, n)	48/16	1125/1333	< 0.001
ULBT > 2 grade (Yes/No, n)	18/46	121/2337	< 0.001
Mouth opening (mm)^#^	35(30–38)	40(38–45)	< 0.001
Thyromental distance (mm)^#^	70(60–77)	77(72–83)	< 0.001
Modified Mallampati test > 2 grade (Yes/No, n)	35/29	394/2064	< 0.001

Data are shown as the means ± standard deviation or numbers.

*All patient characteristics were compared using the Mann–Whitney U test for continuous variables and the chi-square or Fisher exact test for categorical variables.

^#^Data are displayed as the median (interquartile range).

*ULBT* upper lip bite test.

### Predicting difficult intubation

**Figure 2:**
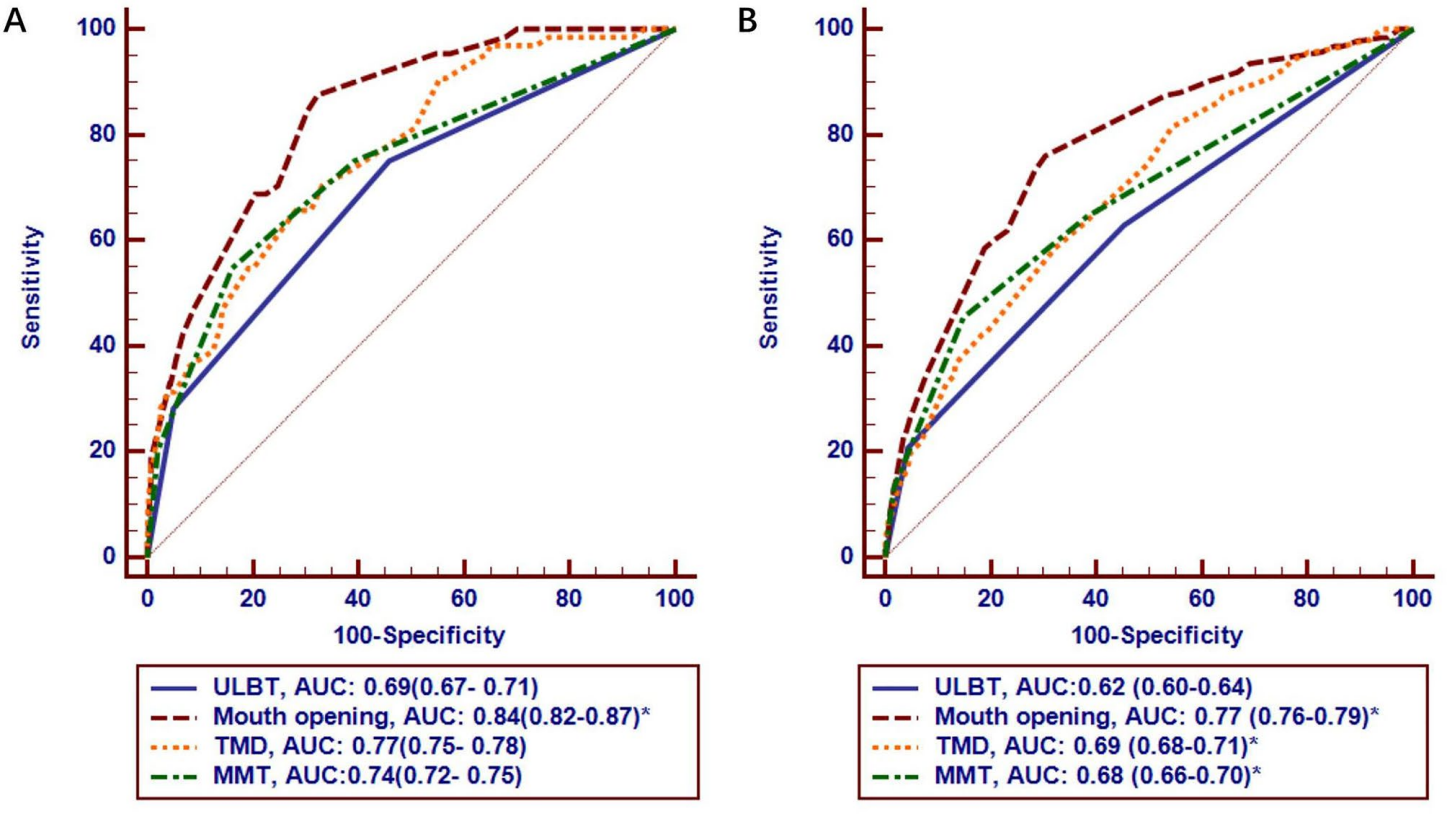
Receiver operating characteristic curve analysis of airway assessment tests and their areas under the curve (AUCs; value and its 95% confidence interval) for predicting difficult intubation (**A**) and difficult laryngoscopy (**B**). *: The difference is significant when compared with the AUC of the ULBT (*P* < 0.05). Abbreviations: ULBT, upper lip bite test; MMT, modified Mallampati test; TMD, thyromental distance.

The statistical values of each parameter for predicting difficult intubation are shown in Table 2. A grade 2 or higher in the ULBT predicted difficult intubation with a sensitivity of 0.75 (95% CI 0.63–0.85) and a specificity of 0.54 (95% CI 0.52–0.56). A grade 3 in the ULBT predicted difficult intubation with a sensitivity of 0.28 (95% CI 0.18–0.41) and a specificity of 0.95 (95% CI 0.94–0.96). The receiver operating characteristic curve analysis showed that the AUC of ULBT to predict difficult intubation was significantly lower than that of mouth opening (0.69 [95% CI 0.67–0.71] vs. 0.84 [0.82–0.85], *P* < 0.001, Fig. 2A).

**Table 2:** Variable values to predict difficult intubation (n = 2522).

Variables	Adjusted odds ratio (95% CI)	Sensitivity (95% CI)	Specificity (95% CI)	PPV (95% CI)	NPV (95% CI)
Age > 52 y	1.9(1.1–3.3)	0.63(0.50–0.74)	0.60(0.58–0.62)	0.04(0.03–0.05)	0.98(0.98–0.99)
ULBT > 2 grade	3.0(1.6–5.6)	0.28(0.18–0.41)	0.95(0.94–0.96)	0.13(0.09–0.19)	0.98(0.98–0.98)
Mouth opening < 4.0 cm	7.1(3.5–14.4)	0.84(0.73–0.92)	0.70(0.68–0.72)	0.07(0.06–0.08)	0.99(0.99–1.00)
Thyromental distance < 7.0 cm	2.6(1.5–4.4)	0.55(0.42–0.67)	0.81(0.79–0.82)	0.07(0.06–0.09)	0.99(0.98–1.00)
Modified Mallampati test > 2 grade	3.3(1.9–5.6)	0.55(0.42–0.67)	0.84(0.83–0.85)	0.08(0.07–0.10)	0.99(0.98–1.00)

*CI* confidence interval, *NPV* negative predictive value, *PPV* positive predictive value.

### Predicting difficult laryngoscopy

The statistical values of each parameter for predicting difficult laryngoscopy are shown in Table 3. A grade 2 or higher in the ULBT had a sensitivity of 0.63 (95% CI 0.55–0.70) and a specificity of 0.55 (95% CI 0.53–0.57); a grade 3 in the ULBT had a sensitivity of 0.21 (95% CI 0.15–0.27) and a specificity of 0.96 (95% CI 0.95–0.97). The AUC of ULBT for predicting difficult intubation was 0.62 (95% CI 0.60–0.64), which was significantly lower than that of mouth opening, thyromental distance, and the modified Mallampati test, as shown in Fig. 2B.

**Table 3:** Variable values to predict difficult laryngoscopy (n = 2522).

Variables	Adjusted odds ratio (95% CI)	Sensitivity (95% CI)	Specificity (95% CI)	PPV (95% CI)	NPV (95% CI)
Age > 52 y	1.5(1.1–2.1)	0.56(0.48–0.63)	0.61(0.59–0.63)	0.10(0.08–0.12)	0.95(0.93–0.96)
ULBT > 2 grade	2.9(1.8–4.1)	0.21(0.15–0.27)	0.96(0.95–0.97)	0.27(0.21–0.35)	0.94(0.94–0.94)
Mouth opening < 4.0 cm	4.6(3.2–6.6)	0.73(0.66–0.80)	0.72(0.70–0.74)	0.17(0.15–0.18)	0.97(0.96–0.98)
Thyromental distance < 7.0 cm	1.9(1.3–2.6)	0.42(0.35–0.50)	0.82(0.80–0.83)	0.15(0.13–0.18)	0.95(0.94–0.95)
Modified Mallampati test > 2 grade	2.9(2.1–4.1)	0.45(0.38–0.53)	0.85(0.84–0.87)	0.19(0.16–0.22)	0.95(0.95–0.96)

*CI* confidence interval, *NPV* negative predictive value, *PPV* positive predictive value, *ULBT* upper lip bite test.

### Multivariate logistic regression analysis

The collinearity test found that there is no collinearity among the variables in Table 1 (Variance inflation factor < 10). Variables with significant differences in Table 1 were included in multifactor logistic regression analysis. The results showed that when predicting difficult intubation or predicting difficult laryngoscopy, ULBT (> 2 grade), age (> 52 years old), M Testing (> 2 grade), mouth opening (< 40 mm), and thyroid-mental distance (< 70 mm) were independent predictors. Their corresponding predicting parameters are shown in Tables 1 and 2. The area under the ROC curve of the multi-factor combination model for predicting difficult intubation was 0.88 (95% CI 0.86–0.89), and the area under the ROC curve for predicting difficult laryngoscopy was 0.81 (95% CI 0.79–0.82).

## Discussion

In this study, a sample size of more than 2000 cases and more than 60 patients with difficult intubation provided a basis for verifying the performance of the ULBT in predicting difficult intubation. The results of this study showed that even though the ULBT was significantly effective in predicting difficult intubation but had no clinical advantage. Overall, the AUC reached 0.69 for predicting difficult intubation, reached 0.62 for predicting difficult laryngoscopy. When compared with the commonly used mouth opening test, it was found that the ULBT was inferior in predicting both difficult intubation and difficult laryngoscopy. This result is similar to previous results^14,15,27^.

In terms of sensitivity and specificity, the results are quite different^2,28^. However, it should be noted that these changes may be an effect of observations based on different cutoff values. The data of this study showed that in a grade 2 ULBT, the sensitivity was higher and its specificity was lower, and in a grade 3 ULBT, the sensitivity was lower but its specificity was higher. Limited by overall predictive performance, it seems difficult to balance sensitivity and specificity. However, there is generally accepted method to determine the threshold value. For Youden's index, sensitivity and specificity are both considered. Previous studies^15,27^ showed that the Youden’s index was optimal when grade 2 was used as the threshold, which is similar to present data, but the specificity was low in this situation.

Similar to previous findings^27^, in the present observation, the predictive performance of the ULBT for difficult airway was not superior to that of mouth opening. Temporomandibular joint mobility assessment may be the main purpose of the ULBT. All movement of the temporomandibular joint depends on the downward rotation of the mandible and the forward and downward sliding of the condyles^29^. Since there is no need to open the mouth during the ULBT method, there is no downward rotation of the mandible, which limits the sliding distance of the temporomandibular joint condyle. This may be the reason why the ULBT does not adequately reflect the range of motion of the temporomandibular joint.

We note in Table 1 that there is a significant difference in age when comparing patients with difficult intubation to those without. Kheterpal et al.^30^ have confirmed that age is an independent risk factor for difficult mask ventilation. The data from this study also indicate that age impacts the difficulty of tracheal intubation. However, the specific mechanisms by which age influences the development of difficult airways remain unclear. Generally, the formation of a difficult airway is associated with age-related anatomical changes in the airway and alterations in joint function. Nevertheless, the age-related factors contributing to these changes require further elucidation.

The effectiveness of any single factor in predicting a difficult airway is limited; thus, using a combination of factors could potentially improve predictive performance. Results from the multivariate logistic regression analysis indicated that the ULBT > 2 grade, rather than ULBT > 1 grade, was an independent risk factor for difficult intubation. The AUC of the combined model—which includes all independent predictors of difficult intubation—reached 0.88. However, when compared with the AUC of 0.84 for the single factor of mouth opening, the combined model does not show a significant advantage in predictive performance.

This study also has some limitations. There is still an insufficient sample size to investigate difficult mask ventilation; the effect of the ULBT in combination with other methods in predicting difficult airway was not investigated; also lack of data for analysis of differences between different races. In the absence of complete muscle relaxation monitoring, patients’ intubation conditions might not be uniform. Although we tried to include experienced operators in the study, the experience of the endotracheal intubation provider may have some influence on the results.

In summary, the ULBT lacks significant clinical advantages in predicting difficult intubation, and more effective methods need to be further explored.

## Data Availability

Datasets of this study can be accessed by request from the corresponding author.

## Abbreviations

ULBT: Upper lip bite test
CI: Confidence intervals
ROC: Receiver operating characteristic curve
AUC: Area under the receiver operating characteristic curve
NPV: Negative predictive value
PPV: Positive predictive value
MMT: Modified Mallampati test
TMD: Thyromental distance
